# Effects of Interfacial Adhesion on Lithium Plating Location in Solid‐State Batteries with Carbon Interlayers

**DOI:** 10.1002/adma.202502114

**Published:** 2025-05-12

**Authors:** Daniel W. Liao, Davy Zeng, Muzamil Mulla, Ali Madanchi, Hiroki Kawakami, Yuichi Aihara, Koichiro Aotani, M. D. Thouless, Neil P. Dasgupta

**Affiliations:** ^1^ Department of Mechanical Engineering University of Michigan Ann Arbor MI 48109 USA; ^2^ Department of Materials Science and Engineering University of Michigan Ann Arbor MI 48109 USA; ^3^ Nissan Research Center Nissan Motor Co. Ltd. Natsushima Yokosuka Kanagawa 237‐8523 Japan

**Keywords:** adhesion, anode‐free, carbon interlayer, lithium metal anode, mechanical properties, solid‐state battery

## Abstract

Carbon interlayers have been implemented in “anode‐free” solid‐state batteries to improve the uniformity and reversibility of lithium deposition by controlling the location of Li plating. However, there remains a lack of fundamental understanding of the detailed role of how these interlayers function during in situ Li formation. In this study, the relationships between the interfacial adhesion of the carbon interlayer to the solid electrolyte and the location of Li plating are investigated. By varying the lamination pressure used during manufacturing, the ability to systematically tune the resulting interfacial adhesion is demonstrated. Mechanical peel tests are performed, and a 4‐fold increase in interfacial toughness is measured as the lamination pressure increases from 100 to 400 MPa. Post‐mortem electron microscopy revealed that the location of Li plating with respect to the carbon interlayer transitions from the interface with the solid electrolyte to the current collector above a threshold interfacial toughness, which is consistent when the interlayer material is changed from amorphous to hard carbon. These findings highlight the role of electro‐chemo‐mechanical relationships in systematically controlling Li deposition in solid‐state batteries when interlayers are present.

## Graphical abstract

Carbon interlayers can promote the stable cycling of anode‐free solid‐state batteries, but their underlying mechanisms have remained elusive. In this study, a direct correlation is observed between the interfacial adhesion of the carbon interlayer and the location of lithium plating. By conducting quantitative peel tests, a threshold interfacial toughness is found to be a determining factor that directs plating toward specific interfaces.

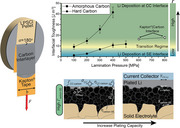

## 1 Introduction

Solid‐state batteries (SSBs) have been proposed as a safer alternative to traditional Li‐ion batteries (LIBs), by replacing the flammable liquid electrolyte with a solid electrolyte (SE). Additionally, SSBs can realize a step increase in energy density by using a lithium (Li) metal anode to replace the traditional graphite anode material.^[^ ^1^ ^]^ Recently, increasing attention has been focused on “anode‐free” cell configurations, where the Li metal negative electrode is formed in situ during the initial charge step. This allows for further increases in volumetric and gravimetric energy density and simplifies the manufacturing process due to the absence of excess Li.^[^ ^1^, ^2^, ^3^ ^]^ However, several challenges remain in the implementation of anode‐free configurations, including inhomogeneous Li nucleation,^[^ ^4^ ^]^ solid electrolyte interphase (SEI) formation,^[^ ^5^ ^]^ dendrite/filament formation,^[^ ^6^ ^]^ Li isolation during stripping,^[^ ^7^, ^8^ ^]^ and inconsistent interfacial contact that can result in reduced cycle life.^[^ ^9^ ^]^

The relatively rigid nature of the interface between Li metal and the solid electrolyte in SSBs presents unique attributes and challenges in comparison to that of a liquid electrolyte.^[^ ^10^ ^]^ For example, the solid‐solid point contacts along the interface limit the electrochemically active area by restricting ionic and electronic pathways.^[^ ^11^, ^12^, ^13^, ^14^ ^]^ The point contacts can also result in local stress concentrations that can lead to the eventual fracture of ceramic SEs, particularly during continuous cycling in the presence of large volume changes.^[^ ^15^ ^]^ The dynamic evolution of these mechanical phenomena during both plating and stripping can result in further changes in the interfacial contact area, which has significant influences on the interfacial electrochemistry and degradation mechanisms.^[^ ^16^, ^17^, ^18^ ^]^ Hence, there remains a need to maintain conformal contact between the electrode and SE during cycling to improve the uniformity of both electrical and ionic conduction across the interface, while also sustaining the dynamic volumetric changes that occur during cell operation.

Recent efforts have introduced interlayers between the current collector (CC) and the solid electrolyte that modify the Li nucleation and growth processes by affecting the wetting, ionic diffusivity, elastic modulus, and strain rate along the interfacial region, with the goal of maintaining intimate contact between the Li metal anode and the solid electrolyte.^[^ ^19^, ^20^, ^21^, ^22^, ^23^, ^24^ ^]^ For example, reactive metals such as Ag, Au, Mg, and In have been implemented to achieve a uniform and dense formation of Li metal during the initial formation cycle.^[^ ^19^, ^25^, ^26^ ^]^ However, interlayers that alloy with Li may not remain adhered to the initial interface after multiple cycles, which may reduce their efficacy during long‐term cycling.^[^ ^25^, ^27^, ^28^ ^]^ To address this challenge, porous carbon‐silver (Ag) interlayers have been investigated as an alternative material system that can sustain the volumetric changes from Li plating and stripping, while maintaining intimate contact across the SE interface.^[^ ^29^ ^]^ These carbon interlayers have received significant attention due to their long‐term efficacy in relevant solid‐state pouch cell formats.^[^ ^29^, ^30^, ^31^ ^]^

A variety of carbon interlayer compositions and alloy additives have been investigated in anode‐free SSBs, which have been fabricated using a range of manufacturing techniques.^[^ ^29^, ^32^, ^33^, ^34^, ^35^ ^]^ These studies have observed differences in the location of Li plating, which can occur either at the interface between the current collector and carbon, or between the carbon interlayer and the solid electrolyte.^[^ ^29^, ^31^, ^33^ ^]^ Owing to these observed differences in plating behavior, there have been an increasing number of efforts that aim to understand the underlying mechanisms that guide the associated nucleation and growth processes.

A commonly proposed mechanism involves Li diffusion through the carbon interlayer, with concomitant lithiation of the carbon. Eventually, the carbon “host material” will supersaturate, and metallic Li will precipitate out of the carbon.^[^ ^32^, ^33^, ^36^, ^37^, ^38^, ^39^, ^40^, ^41^ ^]^ If a secondary Ag phase is present in the interlayer, subsequent alloy formation will occur, which promotes improved wetting and homogeneity of the deposited Li.^[^ ^37^ ^]^ However, it has been shown that Li plating can occur at the interface between the carbon and current collector even without an alloying Ag phase present, demonstrating that the origins of this plating location are fundamentally related to the presence of carbon in the system.^[^ ^36^ ^]^

The transition in reaction pathways from lithiation of the carbon layer to Li plating has been shown to be dependent on the applied current density. These rate‐dependent phenomena can influence the formation of Li concentration gradients and subsequent relaxation dynamics, which alter the initial Li nucleation and growth behavior.^[^ ^36^ ^]^ For example, as current density increases, the amount of charge capacity that is passed before the nucleation peak in the voltage trace decreases, indicating that the total capacity of the carbon interlayer does not necessarily have to be lithiated prior to nucleation and growth of Li toward the CC interface.^[^ ^36^ ^]^ In addition, the particle sizes of the carbon and SE phases have been shown to play a role in Li creep behavior^[^ ^42^ ^]^ and deposition morphology,^[^ ^43^ ^]^ which can influence the transport of plated Li toward either the interface between the CC and carbon, or between the carbon and SE.

In addition to these chemical and electrochemical factors, recent work has also proposed that the adhesion between the two interfaces that are formed with the carbon interlayer (at the SE and CC sides of the interlayer, respectively) can play a role in determining the location of Li plating. It has been proposed that the weaker interface is more susceptible to detachment from the carbon interlayer, which could facilitate Li growth at this location.^[^ ^31^, ^39^, ^42^ ^]^ However, there is a lack of quantitative experimental data on the mechanical properties of these interfaces, which has limited our mechanistic understanding of the role that interfacial mechanics plays in determining the location of Li deposition. Knowledge of these mechanical properties is also needed to guide future computational models that aim to design and optimize interlayers for SSBs.

In this work, we explore the relationships between the interfacial mechanics of the carbon interlayer on a solid electrolyte and the resulting Li plating behavior, by systematically varying the interfacial adhesion. Using a quantitative peel test to measure interfacial toughness values, we demonstrate that when the lamination pressure is increased from 100 to 400 MPa, the interfacial toughness between the carbon and SE increases by a factor of four. We observe a direct correlation between the interfacial toughness and location of Li plating, where Li plating preferentially occurs at the interface between the CC and carbon only after the interfacial toughness between the carbon and the SE exceeds a critical threshold of ≈10 J m^−2^. A similar threshold value in interfacial toughness was observed when the type of carbon and particle size were changed from amorphous to hard carbon. Overall, these findings illustrate the importance of interfacial mechanics as a critical factor in controlling the location of Li plating, which will be determined by consideration of the overall energy balance of the system.

## 2 Results and Discussion

**Figure 1:**
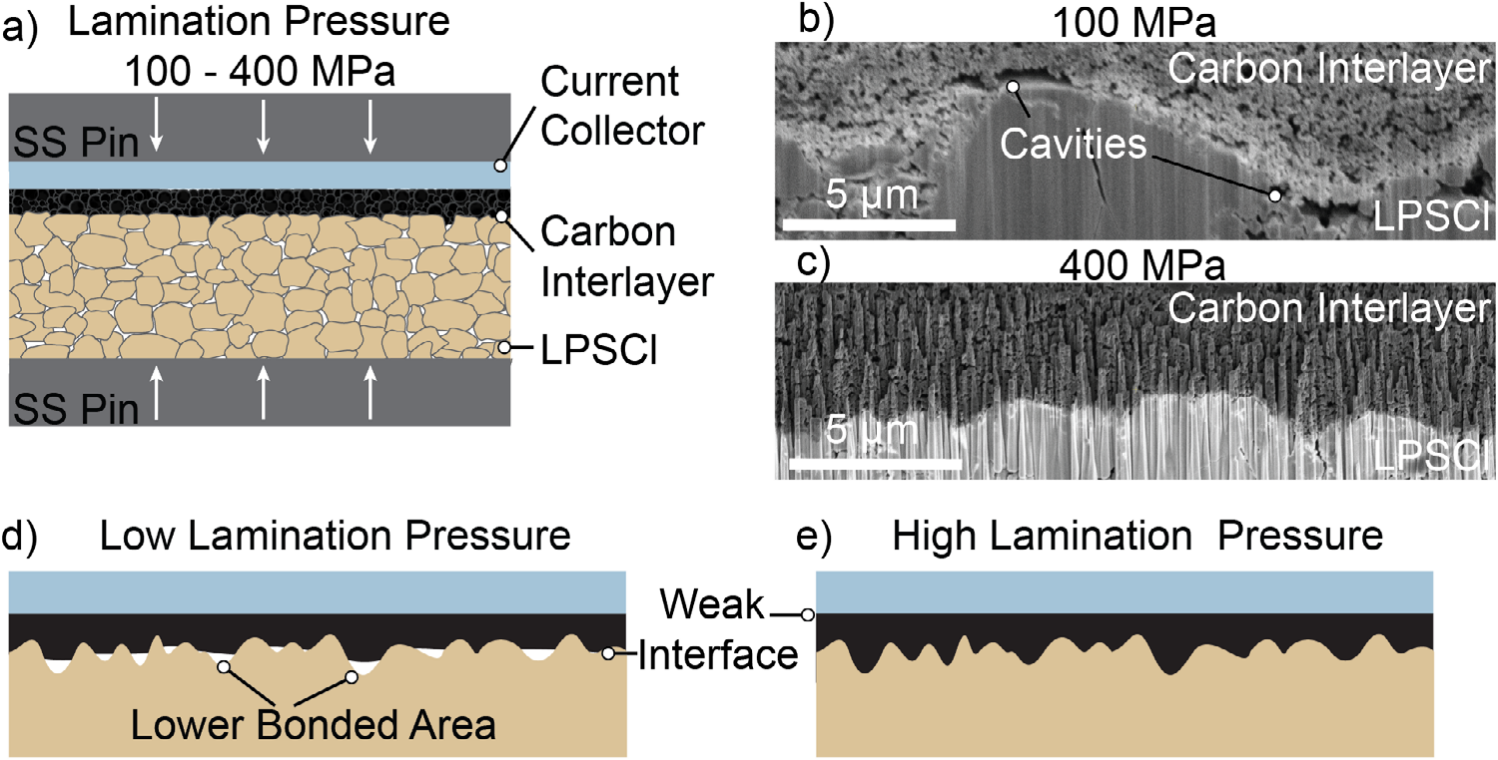
a) Schematic of the lamination procedure used to adhere the carbon interlayer onto the SE. b,c) Cross‐sectional plasma‐focused ion beam‐scanning electron microscopy images of the interface between the carbon interlayer and the SE using lamination pressures of (b) 100 and (c) 400 MPa. d,e) Schematic of the resulting interfacial morphology of the carbon interlayer after (d) low and (e) high lamination pressures.

To control the interfacial adhesion between the carbon interlayer and solid electrolyte during cell manufacturing, a lamination approach was employed (**Figure** **1**a). To fabricate the carbon interlayer, a slurry of amorphous carbon with an average particle size of ≈30 nm was first cast onto a stainless‐steel foil to fabricate a ≈13 µm thick carbon interlayer. The casted amorphous carbon interlayer was then cut out and pressed onto a 6 mm diameter Li_6_PS_5_Cl (LPSCl) pellet by applying uniaxial compression. To control the interfacial adhesion between the carbon and SE, a range of lamination pressures (400, 300, 200, and 100 MPa) were used. After lamination, the stainless‐steel foil that the carbon slurry was cast on can be easily removed, resulting in a complete transfer of the carbon interlayer onto the solid electrolyte. Further details on the fabrication procedures can be found in the Experimental Section.

We observe a difference in the morphology of the interface as a function of lamination pressure (Figure 1b,c). At a low lamination pressure (100 MPa), we observe a lower bonded area along the interface, where microscopic cavities are present (Figure 1b). In contrast, at a higher lamination pressure (400 MPa), the carbon interlayer forms a more conformal interface with the SE (Figure 1c). These differences in the conformality of the interfacial contact are a result of greater plasticity at elevated lamination pressures. As a consequence of this improved contact, the interfacial adhesion is significantly greater at higher lamination pressures (Figure 1d,e), which will be quantitatively demonstrated below. We hypothesize that the presence of these interfacial cavities, and the associated decrease in interfacial adhesion, will influence the nature of Li nucleation and growth, which has been previously observed in anode‐free SSBs without carbon interlayers.^[^ ^44^ ^]^

**Figure 2:**
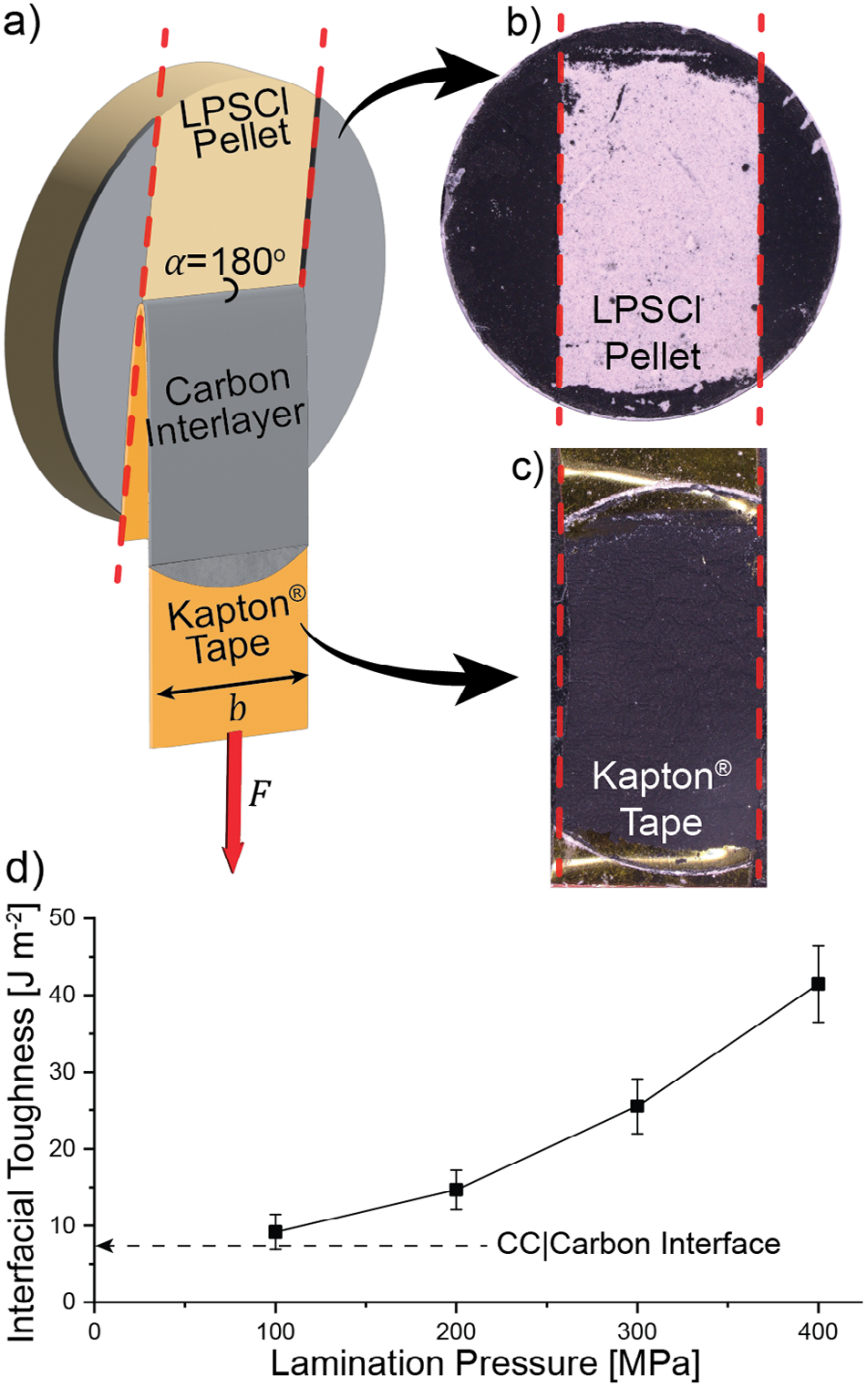
a) Schematic of the peel‐test geometry, where Kapton tape was used to delaminate the carbon interlayer from an LPSCl solid electrolyte pellet. Top‐down optical microscopy images of the b) LPSCl pellet and c) Kapton tape after a peel test, when the amorphous carbon interlayer was laminated onto the SE at 300 MPa. d) Measured interfacial toughness values at various lamination pressures for amorphous carbon interlayers. The error bars represent independent measurements from *n* = 3 samples. The horizontal dashed arrow represents the measured interfacial toughness of the carbon interlayer and stainless‐steel current collector after the initial casting and drying processes.

To quantify the interfacial adhesion between the carbon interlayer and solid electrolyte, a systematic series of peel tests were conducted (**Figure** **2**). While an earlier study reported qualitative differences in adhesion between the carbon interlayer and SE using a peel test,^[^ ^42^ ^]^ to the best of our knowledge, our work is the first to systematically vary and experimentally quantify the interfacial toughness of carbon interlayers adhered on a SE. After the lamination process, the current collector was removed and a Kapton tape was adhered to the carbon interlayer. The tape was then peeled off at a peel angle (α) of 180° with respect to the substrate surface (Figure 2a).

For an inextensible tape in the absence of plasticity, the toughness of the interface can be directly computed from the peel force.^[^ ^45^ ^]^ The validity of this approach requires the elastic strain energy to be negligible,^[^ ^46^ ^]^ and the interface to be sufficiently brittle that delamination can occur without plastic bending.^[^ ^47^, ^48^ ^]^ In this study, it was found that plastic deformation of the Kapton tape occurs at larger peel forces (Figure S1, Supporting Information). Therefore, an additional analysis was developed to incorporate these effects when calculating the interfacial toughness (Table S1, Supporting Information), which is further discussed in the Supporting Information.

After the peel test, optical microscopy was performed to determine the location where delamination occurred (Figure 2b,c). For the range of lamination pressures applied in this study (100–400 MPa), delamination was observed to consistently occur between the carbon interlayer and SE interface, resulting in the transfer of the carbon interlayer from the SE onto the Kapton tape. The fracture across the interface between the carbon interlayer and SE interface was rapid, resulting in the complete removal of the carbon interlayer across the length of the LPSCl pellet.

The average interfacial toughness was calculated based on measurements from *n* = 3 independent samples for each lamination pressure (Figure 2d). The interfacial toughness between the carbon and SE increases monotonically with increasing lamination pressure, ranging from 9 ± 2 J m^−2^ at a pressure of 100 MPa, to 41 ± 5 J m^−2^ at a pressure of 400 MPa. This is consistent with the SEM analysis shown in Figure 1b,c, where the interfacial bonding was more continuous at higher lamination pressures.

An additional peel test was performed to measure the interfacial adhesion between the carbon interlayer and the stainless‐steel current collector after the initial casting and drying process (prior to lamination). The interfacial toughness was measured to be 7 ± 1 J m^−2^, which is shown as the horizontal dashed arrow in Figure 2d. This value serves as a lower limit for the interfacial toughness values that we were able to measure between the amorphous carbon interlayer and the SE. During lamination, the adhesion between the carbon and SE must exceed this value to transfer the carbon onto the solid electrolyte surface. This is consistent with the observation that all of the measured interfacial toughness values between the carbon and solid electrolyte after lamination were greater than 7 J m^−2^ (Figure 2d).

To determine the location of Li plating with respect to the carbon interlayer for different interfacial toughness values, cross‐sectional plasma‐focused ion beam–scanning electron microscopy (PFIB‐SEM) was performed. To ensure that metallic Li supersaturates and precipitates out of the carbon interlayer, cells were charged to a capacity of 2 mAh cm^−2^, which is significantly higher than the lithiation capacity of the amorphous carbon material (≈0.25 mAh cm^−2^).^[^ ^36^ ^]^ A Xe PFIB was used to perform cross‐sectional milling to minimize reactivity between the ion beam and the plated Li metal.^[^ ^49^ ^]^ Further details on the plating process are provided in the Experimental Section.

**Figure 3:**
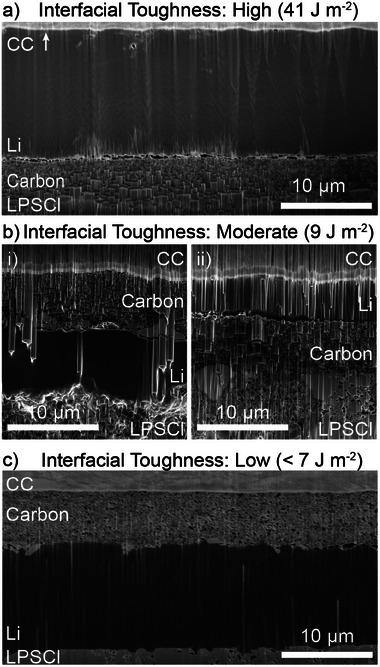
Cross‐sectional PFIB‐SEM images after Li plating with a capacity of 2.0 mAh cm^−2^ at a current density of 0.1 mA cm^−2^ for amorphous carbon interlayers that were laminated onto the SE at a) 400 MPa, b) 100 MPa, and c) 5 MPa. The corresponding interfacial toughness values between the carbon and SE are also indicated at each lamination condition.

When a lamination pressure of 400 MPa was used, Li deposition was only observed at the interface between the CC and carbon interlayer (**Figure** **3**a). PFIB cross‐sections were performed at multiple locations across the current collector to confirm this trend (Figure S2, Supporting Information). This plating behavior is consistent with previous reports that have used high lamination pressures to form a well‐adhered and conformal interface between the carbon and SE.^[^ ^29^, ^31^, ^36^ ^]^ At an even higher lamination pressure, 600 MPa, the Li was also observed to deposit at the interface between the CC and carbon interlayer (Figure S3, Supporting Information). These results illustrate that when the adhesion at the carbon and SE interface is sufficiently strong, Li deposition preferentially occurs at the interface between the CC and carbon.

When the lamination pressure was reduced to 100 MPa, the plated Li was no longer observed to occur exclusively at the interface between the CC and carbon. In some locations, Li plating occurred at the interface between the CC and carbon, while at other locations, Li plating was observed at the interface between the carbon and SE (Figure 3b). The presence of Li at the interface between the carbon and SE for a lamination pressure of 100 MPa is correlated with the lower interfacial toughness at this condition (Figure 2d). These observations suggest that the ability of Li to plate at this interface is influenced by how easily the carbon interlayer can delaminate from the solid electrolyte surface. The fact that plated Li was observed at both interfaces when the lamination pressure was 100 MPa further suggests that the Li plating mechanism is affected by spatial inhomogeneity along the interface between the carbon and SE. An example of this can be observed in Figure 1b, where a more discontinuous interface was observed when the lamination pressure was decreased to 100 MPa.

In some locations across the sample that was laminated at 100 MPa, we observed cohesive fracture of the carbon interlayer, which resulted in a transition between plating at the interface with the solid electrolyte and plating at the current‐collector interface (Figure S4, Supporting Information). At these moderate lamination conditions, where the bonded area along the interface with the SE is lower (Figure 1b), the nucleation and growth of Li will be influenced by the discontinuities along the interface. In particular, the presence of microscopic cavities along the interface between the carbon and SE can promote preferential nucleation in these locations to fill the preexisting cavities during the early stages of plating (Figure S5, Supporting Information). The preferential nucleation and growth of Li within microscopic cavities along the anode‐free interface between a metal CC and SE was also observed in a previous study, where no interlayer was present.^[^ ^44^ ^]^ This spatial inhomogeneity in the plated Li morphology will result in localized bending and tensile stresses within the carbon interlayer, which could promote cohesive fracture of the carbon.

To intentionally create an even weaker interface between the carbon and SE, the as‐cast carbon interlayer on the current collector was directly pressed onto an LPSCl pellet at a stack pressure of 5 MPa, without any prior lamination. Under this condition, the carbon interlayer remains adhered to the CC and does not transfer to the solid electrolyte surface prior to the initial charge step (Figure S6, Supporting Information). As discussed above, the measured interfacial toughness between the CC and carbon interlayer was 7 ± 1 J m^−2^, which suggests that the adhesion between the carbon and SE at a 5 MPa stack pressure was lower than this value, because the transfer of the carbon interlayer onto the SE does not occur. Under these conditions of low interfacial adhesion (relative to the interface between the CC and carbon), Li was observed to exclusively deposit at the interface between the carbon interlayer and SE (Figure 3c). Therefore, in this study, we did not further pursue methods to further increase the interfacial toughness between the CC and carbon interlayer, as this promotes undesirable Li deposition toward the solid electrolyte.

When considering the physics of the system, at first glance, one might intuitively expect Li to always plate at the interface between the carbon and SE, because there is an additional energy penalty required for Li to transport through the carbon interlayer to reach the current collector. For mixed electronic‐ionic conductors such as carbonaceous materials, where the electronic conductivity is significantly higher than the ionic conductivity, the ohmic losses associated with ionic conduction are expected to be larger than those for electronic conduction, which would favor Li plating at the interface between the carbon and SE.^[^ ^39^, ^40^, ^50^ ^]^ It has also been proposed that Li plating initiates within the pores of the carbon interlayer, and subsequently extrudes to the interface with weaker adhesion.^[^ ^31^, ^39^, ^42^ ^]^ Therefore, it is important to also examine the mechanical contributions to the overall energy balance of the system, when considering the location where Li plating will be most favorable.

As we have previously shown, carbon interlayers in anode‐free SSBs initially lithiate at potentials that are more positive than 0 V versus Li/Li^+^ before a transition in the reaction pathway occurs to initiate Li nucleation as the potential drops below 0 V.^[^ ^36^ ^]^ In that sense, the precipitation of the metallic Li phase is analogous to Li plating on graphite during fast charging of Li‐ion batteries in liquid electrolytes, where a supersaturation and precipitation process occurs.^[^ ^51^ ^]^ Following this analogy, we have further shown that state‐of‐charge gradients form and subsequently relax during high‐rate charging of the carbon interlayer.^[^ ^36^ ^]^ However, despite the presence of these concentration gradients within the carbon interlayer, the location of Li plating was always observed to be at the interface between the carbon and current collector, regardless of the current density and state of charge of the carbon.^[^ ^36^ ^]^ In contrast, when the same carbon interlayer was charged in a liquid electrolyte, Li plating primarily occurred at the opposite interface, between the carbon and the separator (Figure S7, Supporting Information). Together, these results suggest that when the adhesion at the interface between the carbon interlayer and SE is sufficiently high, transport phenomena alone are unlikely to explain the preferential plating of Li at the current collector in the solid‐state systems.

In addition to these transport considerations, there are also energy barriers associated with the nucleation and growth of plated Li, which must be considered in the overall energy balance of the system. In particular, if Li plating were to occur at the interface between the carbon and the SE, there would be an energy barrier associated with the buildup of mechanical stress and subsequent local delamination of the carbon interlayer from the solid electrolyte around the regions where Li deposition occurs.^[^ ^3^, ^52^ ^]^ These phenomena were recently explored in a metal CC that was diffusion bonded to a Li_7_La_3_Zr_2_O_12_ (LLZO) SE to form an anode‐free interface.^[^ ^44^ ^]^ Owing to the strong adhesion between the CC and SE in the diffusion‐bonded system, a significant increase in the hydrostatic stress within the Li was needed to drive delamination of the current collector, which was demonstrated through a combination of operando optical microscopy and electrochemical analysis.^[^ ^44^ ^]^ The energy penalty associated with delamination increases as the interfacial toughness becomes larger, highlighting the importance of quantitatively exploring the relationships between adhesion and Li nucleation and growth.

**Figure 4:**
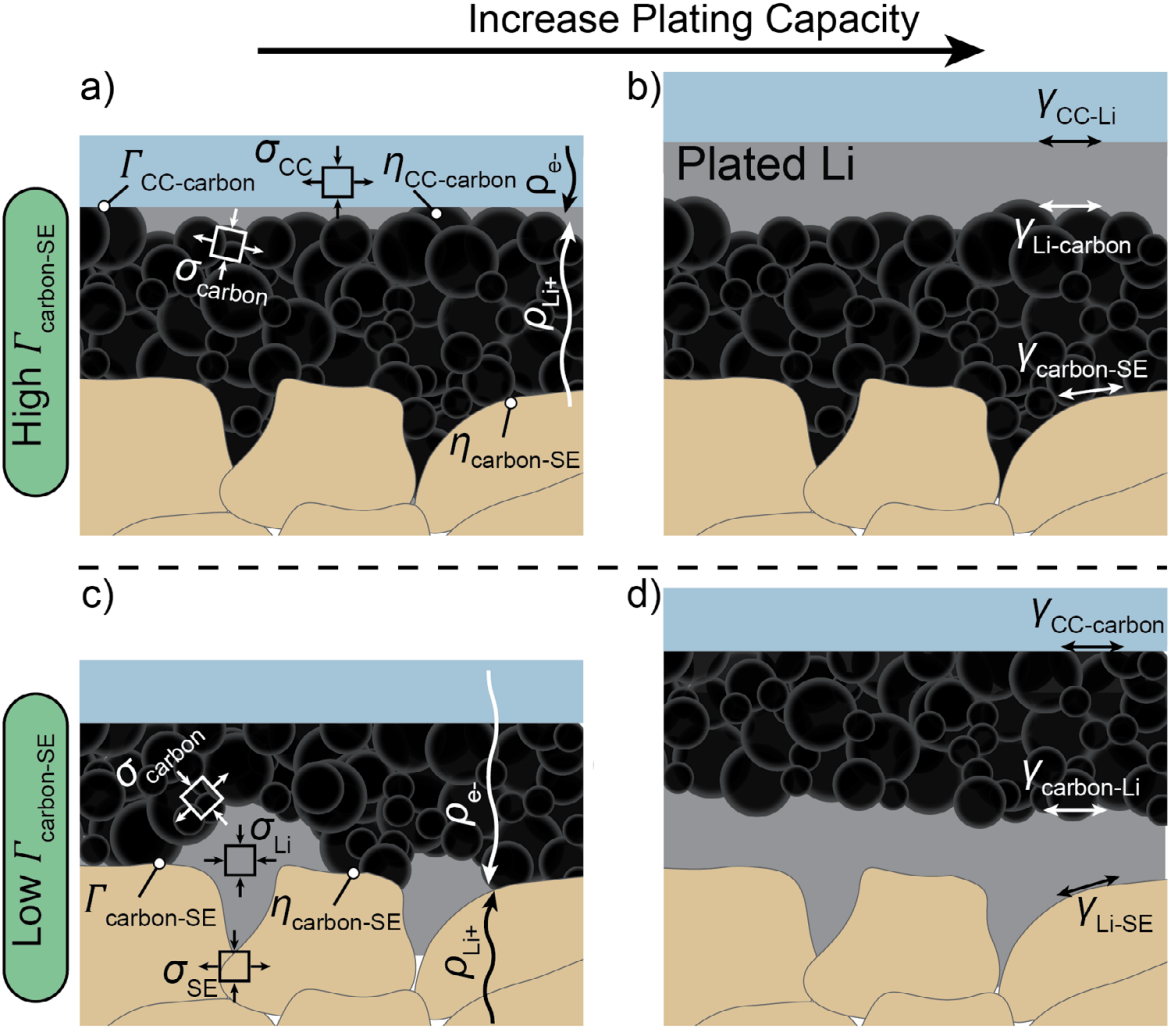
Schematic of the negative electrode region during charging to low and high capacities. a) Initial plating behavior for an interface with high toughness between the carbon and SE. Various elements in the energy balance are depicted, including the Li (*ρ*_Li+_) and electronic (*ρ*_e‐_) resistivities of the carbon interlayer, interfacial toughness between the CC and carbon (*Γ*_CC‐carbon_), overpotentials at the interfaces between the carbon interlayer and the CC and SE (*η*_CC‐carbon_ and *η*_carbon‐SE_), and stresses in the CC, carbon, and Li (*σ*_CC_, *σ*_carbon_, and *σ*_Li_). b) As more capacity is passed, Li continues to deposit at the CC interface with the associated interfacial energies (*γ*_CC‐Li_, *γ*_carbon‐Li_, and *γ*_carbon‐SE_). c) Initial plating behavior for an interface with low toughness between the carbon and SE, including additional elements that must be considered in the energy balance such as interfacial toughness between the carbon and SE (*Γ*_carbon‐SE_) and stress in the SE (*σ*_SE_). d) As more capacity is passed, Li continues to deposit at the SE interface with associated interfacial energy between the Li and SE (*γ*_Li‐SE_).

Informed by these previous studies and the results presented herein, we propose that the location of Li plating with respect to the presence of a carbon interlayer is guided by consideration of the overall energy balance between competing factors at the anode‐free interface (shown schematically in **Figure** **4**).^[^ ^30^, ^39^, ^42^, ^44^ ^]^ The major factors that contribute to this energy balance include: 1) the Li^+^ and electronic resistivities within the interlayer and pores (*ρ*_Li+_ and *ρ*_e‐_); 2) the interfacial overpotentials between the carbon interlayer at both the current collector and solid electrolyte (*η*_CC‐carbon_ and *η*_carbon‐SE_); 3) the mechanical stresses present in the various components of the system (*σ*_CC_, *σ*_carbon_ *, σ*_SE_, and *σ*_Li_); 4) the interfacial energies associated with the various contact regions (*γ*_CC‐Li_, *γ*_Li‐carbon_, *γ*_carbon‐SE_, and *γ*_Li‐SE_); and 5) the interfacial toughness values at the locations where delamination occurs (*Γ*_CC‐carbon_ and *Γ*_carbon‐SE_). In addition to these material properties, the geometric parameters of the interlayer (e.g., thickness and porosity) will also influence these terms.^[^ ^41^, ^42^ ^]^

Based on the experimental observations above and the consideration of these energy balances, we hypothesize that when the interfacial toughness between the carbon and SE increases to above a certain threshold value, Li will preferentially deposit toward the current collector (Figure 4a). This threshold will occur when the energy penalty required to delaminate the carbon interlayer from the solid electrolyte becomes sufficiently large, such that other competing factors are insufficient to drive this delamination. As a result, Li growth will preferentially occur at the interface between the CC and carbon (Figure 4b). This is consistent with a previous computational study of plating at the interface between a Li metal anode and SE, which predicted that weak adhesion can lead to interfacial separation.^[^ ^53^ ^]^

While the interfacial toughness will influence the energy barrier required for delamination, the physical driving force for delamination at the interface between the carbon interlayer and SE will be influenced by the mechanical stresses that accumulate in the lithiated carbon and plated Li as it nucleates and grows along the interface. Figure 4c illustrates these relationships for the case where a Li deposit has nucleated and begun to grow at the interface between the carbon and SE. As the plated Li grows larger in size, it will experience compressive stresses (*σ*_Li_) that result from the combination of elastic stresses in the surrounding solid media (*σ*_carbon_, *σ*_SE_), as well as the compressive forces from the externally applied stack pressure.

The spatial distribution of these nucleation and growth processes will be influenced by the nature of the initial interface between carbon and SE. As shown in Figure 1b, when a lower lamination pressure is applied, microscopic cavities are present along the interface between the carbon and SE. As shown schematically in Figure 4c, these cavities act as a triple‐phase boundary where electronic and ionic pathways meet at an empty space. Analogous to the case of a diffusion‐bonded current collector to the SE, these cavities will act as preferential sites for Li nucleation and growth in the early stages of plating, which was observed experimentally using cross‐sectional PFIB‐SEM analysis (Figure S5, Supporting Information). This preferential plating in the cavities arises from a combination of current focusing at the edges of the cavities and the fact that Li can plate into the cavities until they are filled without increasing the pressure that is applied to the surrounding carbon and SE materials.

As growth continues, propagation of a crack‐like layer of Li metal can occur along the interface between the carbon and SE. The energetics of this propagation process will be influenced by the interfacial energy terms. A higher interfacial toughness between the carbon and SE (*Γ*_carbon‐SE_) will make it less energetically favorable for Li metal to propagate out of the nucleation sites along this interface, and will therefore favor plating at the interface between the carbon and CC based on the overall energy balance of the system.

It is noteworthy that this general theory applies to all interfaces in SSBs where micro‐to‐nanoscopic discontinuities will inevitably be present as a result of the manufacturing processes used. The dynamic evolution of these interfacial discontinuities will be influenced by adhesion, Li transport processes (e.g., through migration, diffusion, or creep), the presence of interfacial defects, and the morphology of the interface.^[^ ^53^, ^54^ ^]^ In particular, for porous interlayers such as the carbonaceous materials used in this study, even if there were no microscopic discontinuities present, the pore size itself will factor into this nucleation and growth behavior.^[^ ^43^ ^]^ Overall, these considerations highlight the importance of coupled electro‐chemo‐mechanical phenomena in SSBs, which can inform future models and design criteria for optimized interfaces.

Based on the observations above, we conclude that the location of Li plating with respect to the carbon interlayer is correlated with interfacial toughness and will also be influenced by other factors in the overall energy balance of the system. Therefore, we next examine if these trends can be observed using other carbon‐based porous interlayers. As an alternative model system to the amorphous carbon particles used above, we fabricated and tested interlayers using hard carbon as the active material. Hard carbon has been used previously in LIBs as a high‐rate capability electrode during charging (lithiation) compared to graphite electrodes.^[^ ^55^, ^56^ ^]^ In addition, the hard carbon particles used in our study have a larger diameter (≈3 µm) compared to the amorphous carbon particles (≈30 nm), which will influence the morphology of the interface (Figure S8, Supporting Information). Therefore, the use of this hard carbon interlayer system, which has differing material properties (e.g., ionic conductivity) and geometric parameters (e.g., particle size and porosity) compared to the amorphous carbon used in the experiments described above, provides a second validation on the role of interfacial toughness for Li deposition toward the current collector.

**Figure 5:**
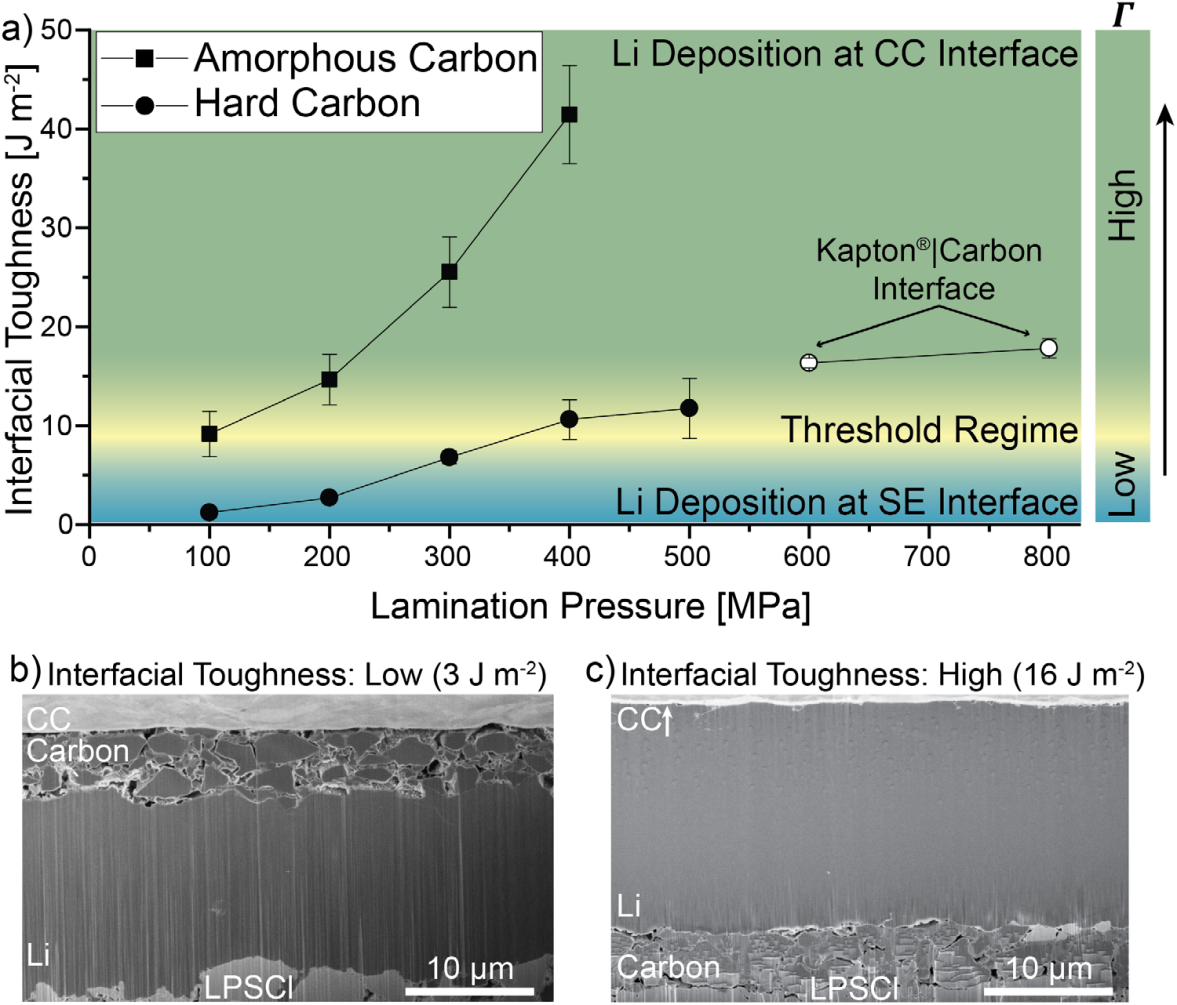
a) Measured interfacial toughness at various lamination pressures for both amorphous and hard carbon interlayers. The error bars represent independent measurements from *n* = 3 samples. The solid data points are samples where delamination occurred between the carbon and SE, and the unfilled data points are samples where delamination occurred between the Kapton tape and carbon. b,c) Cross‐sectional PFIB‐SEM images after Li plating to a capacity of 2.0 mAh cm^−2^ at a current density of 0.1 mA cm^−2^ for hard carbon interlayers at a lamination pressure of (b) 200 and (c) 600 MPa.

Peel tests were performed to quantify the interfacial toughness after laminating the hard carbon interlayer onto the solid electrolyte at varying pressures (100–800 MPa). The measured interfacial toughness for the hard carbon interlayer was lower at each lamination pressure compared to the amorphous carbon (**Figure** **5**a). For example, at a lamination pressure of 400 MPa, the interfacial toughness of the hard carbon (11 ± 2 J m^−2^) is approximately four times lower than that of amorphous carbon (41 ± 5 J m^−2^) at the same lamination pressure. We further note that for very high lamination pressures (>500 MPa), delamination occurred at the interface between the Kapton tape and carbon interlayer during the peel test. This indicates that the adhesion of the interface between the hard carbon and SE was stronger than the interface between the tape and hard carbon at instances when the lamination pressure was sufficiently high. A similar phenomenon was also observed in the amorphous carbon when the lamination pressure was increased to these high values (Figure S9, Supporting Information).

One factor that contributes to the lower interfacial toughness of the hard carbon interlayers in comparison to the amorphous carbon (shown in Figure 5a) is the larger particle size of hard carbon, which reduces the conformality of the carbon interlayer along the laminated interface with the SE (Figure S8, Supporting Information). Additional factors, such as the mechanical, electrochemical, and morphological properties of the two interlayers will also affect the overall energy balance of the system, as depicted in Figure 4. We are therefore interested to see if a similar threshold behavior exists with respect to the relationship between interfacial toughness and the Li plating location.

To evaluate the location of Li plating in the presence of the hard carbon interlayers, PFIB‐SEM analysis was performed. Figure 5b shows an image of Li plating when a relatively low lamination pressure of 200 MPa was used, corresponding to an interfacial toughness of 3 ± 0.5 J m^−2^. Under these conditions, Li plating was exclusively observed at the interface between the carbon and SE. This is consistent with the observations of the amorphous carbon interlayers when the interfacial adhesion between the interlayer and SE was low (Figure 3c). Li plating remained at the same interface when the lamination pressure for hard carbon was increased to 400 MPa (Figure S10, Supporting Information), indicating that the threshold had not yet been reached.

In contrast to these lower lamination pressures for hard carbon, when the lamination pressure was increased to 600 MPa, Li plating was only observed at the interface between the hard carbon and CC. Additional PFIB cross‐sections taken across multiple locations confirm this trend (Figure S11, Supporting Information). This demonstrates that an analogous threshold exists for the hard carbon system, despite the aforementioned differences in the material and geometric properties of the interlayer material. Interestingly, this threshold occurred within a similar range of interfacial toughness for both material systems (≈10 J m^−2^). In Figure 5, the threshold regime is indicated as a yellow band, rather than a singular value, to account for both the experimental uncertainty and the fact that within this range of values, we sometimes observe plating at both interfaces (Figure 3b). Collectively, these results illustrate that while multiple factors will influence the overall energy balance of an interlayer system, interfacial adhesion plays an important role in determining the location of Li plating, where higher adhesion promotes Li plating away from the solid electrolyte for carbon interlayers in anode‐free SSBs.

## 3 Conclusion

In this work, we quantitatively evaluate the role of interfacial toughness on the location of Li plating for anode‐free SSBs with carbon interlayers. We demonstrate that a sufficiently high interfacial toughness at the interface between the carbon interlayer and SE is needed to drive Li plating at the back interface between the CC and carbon. In contrast, when the interfacial toughness is lower than a threshold value of ≈10 J m^−2^, Li plating was observed at the interface between the carbon and SE. This suggests that a threshold of interfacial toughness must be overcome to shift the overall energy balance of the system, which includes additional factors such as the transport properties through the interlayer, interfacial kinetics, mechanical stresses, and interfacial energies.

By comparing two model systems (amorphous carbon vs hard carbon) with multiple differences in their material and geometric properties, we further illustrate that the importance of interfacial toughness in controlling the location of Li plating is not exclusive to one type of carbon material. This study also highlights the importance of understanding the process‐structure relationships during the manufacturing of interlayers in SSBs by revealing the relationships between lamination pressure, interfacial contact, and adhesion. Future work could investigate additional methods to engineer the interfacial adhesion through chemical or physical methods, such as modifying the surface energy or morphology. Overall, these findings highlight the importance of coupled electro‐chemo‐mechanical phenomena in designing and understanding optimal interfaces and interlayers in SSBs. In the future, the quantitative relationships presented in this study could inform future modeling efforts to describe the dynamic evolution of carbon interlayers in anode‐free SSBs.

## 4 Experimental Section

### Fabrication of Carbon Interlayers

To prepare the carbon interlayers, carbon black powder (Li‐100, Denka) or hard carbon (Hard Carbon, Pred. Materials International Inc.) was mixed into a slurry with polyvinylidene fluoride (PVDV) and *N*‐methylpyrrolidone (NMP) using a centrifugal planetary mixer (Thinky Corporation, ARE‐310). The carbon powder and PVDF were mixed at a weight ratio of 86:14. In the case of the amorphous carbon mixture, the slurry was cast onto a stainless‐steel foil. For the hard carbon mixture, the slurry was cast onto a mylar film substrate. The mylar substrate allowed for the hard carbon to effectively transfer onto the SE during the lamination process. The slurry was cast using a screen printer and then dried in air at 80 °C for 20 min and then 100 °C for 12 h. These methods follow the previous work.^[^ ^36^ ^]^

### Fabrication of Solid Electrolyte and Anode‐Free Cell Configuration

All the materials were handled in an inert Ar‐filled glovebox with H_2_O and O_2_ levels below 0.5 ppm. L_6_PS_5_Cl (LPSCl) powders were used as received from MSE Supplies with an average particle diameter of 10 µm. Solid electrolyte pellets (1 mm thick, 6 mm diameter) were first cold pressed to 200 MPa to form a green pellet. Next, the carbon interlayers were laminated onto the surface of the pellets at varying pressures: 800, 600, 500, 400, 300, 200, and 100 MPa. After applying the lamination pressure for 1 min, the carbon layer remained adhered to the solid electrolyte surface and the stainless‐steel foil or mylar substrate can be easily removed. In all the cells, a 10 µm stainless‐steel foil was used as the current collector. The surface roughness of the solid electrolyte along the amorphous carbon interface after lamination was measured to be 0.5–1.0 µm. For the case of the 5 MPa conditions using an amorphous carbon interlayer, the as‐cast carbon on stainless steel foil was directly pressed onto a SE that was previously densified at 400 MPa.

Following previously published procedures,^[^ ^36^ ^]^ bulk Li foil (1.5 mm thick, Thermo Scientific Chemicals) was used as the counter electrode. A spatula was used to scrape the Li foil to remove any surface contamination layer until a shiny metallic luster was observed. The Li foil was then compressed under a pressure of 17.7 MPa to form a thin Li counter electrode. The Li electrode was brought into contact with the solid electrolyte pellet on the opposite side of the carbon interlayer.

The cell assembly was inserted into a PEEK sleeve with an inner diameter of 6 mm. A conductive elastic body was placed between the stainless‐steel current collector and the metal pin that was used to apply stack pressure to ensure uniform pressure distribution across the cell.^[^ ^44^ ^]^ A stack pressure of 5 MPa was used with a constant temperature of 60 °C for the duration of cycling.

### Electrochemical Analysis

After cell assembly, the cell was held at the desired stack pressure and temperature under open circuit voltage (OCV) conditions for 1 h to allow for the cell to equilibrate. An Arbin LBT cycler was used for electrochemical cycling. Galvanostatic charging was applied to form the Li metal anode in situ at a current density of 0.1 mA cm^−2^ for a total plated capacity of 2.0 mAh cm^−2^.

### Materials Characterization

Cross‐sections were prepared with an Xe plasma focused‐ion beam and imaged using scanning electron microscopy with a Thermo Fisher Helios G4 PFIB UXe. PFIB was performed at a current of 0.2 µA and an accelerating voltage of 30 kV. All optical imaging was performed with a Keyence VHX‐7000 4k digital microscope inside an Ar glovebox.

### Peel Test

The peel tests were conducted inside an inert Ar‐filled glovebox. A 3 mm wide Kapton tape was adhered to the carbon interlayers that were laminated onto the LPSCl pellets at varying lamination pressures. The LPSCl pellet was secured vertically such that the tape was peeled off at a 180° angle relative to the substrate by gradually overloading the tape (Figure 2a). A total of three samples per condition were measured to calculate the average and standard deviation of the interfacial toughness. Weights were added incrementally to the tape until delamination occurred.

### Preparation of Liquid Electrolyte and Anode‐Free Cell Configuration

Coin cells were assembled inside a glovebox with H_2_O and O_2_ levels <0.5 ppm. A 14 mm diameter Li metal counter electrode at ≈500 µm thickness was used. 1 m lithium bis(trifluoromethanesulfonyl)imide (LiTFSI) in 1,3‐dioxolane and 1,2‐dimethoxyethane (DOL/DME) with 1 wt.% LiNO_3_ additive was used as the electrolyte and a Celgard polymer was used as the separator. The as‐cast amorphous carbon interlayer onto the stainless‐steel current collector was used as the working electrode. The coin cells were cycled at room temperature under no externally applied stack pressure using a Maccor 4000 series.

### Statistical Analysis

For both the amorphous and hard carbon interlayers at each lamination pressure, a total of three samples were measured to calculate the interfacial toughness mean and standard deviation. The reported interfacial toughness values are presented as mean ± standard deviation. The measured peel force was used to calculate the interfacial toughness as described in the Supporting Information. No additional pre‐processing of the data was performed.

## Conflict of Interest

The authors declare no conflict of interest.

## Supporting information



Supporting Information

## Data Availability

The data that support the findings of this study are available from the corresponding author upon reasonable request.
