# Supplementary material for: Effects of Interfacial Adhesion on Lithium Plating Location in Solid‐State Batteries with Carbon Interlayers
Source: Adv Mater. 2025 May 12;37(29):2502114. doi: 10.1002/adma.202502114 (PMC12288772; doi:10.1002/adma.202502114)
Supplement: Supplementary file 1 — Supporting Information [file ADMA-37-2502114-s001.pdf]

ADVANCED MATERIALS

Supporting Information

for *Adv. Mater.*, DOI 10.1002/adma.202502114

Effects of Interfacial Adhesion on Lithium Plating Location in Solid-State Batteries with Carbon Interlayers

*Daniel W. Liao, Davy Zeng, Muzamil Mulla, Ali Madanchi, Hiroki Kawakami, Yuichi Aihara, Koichiro Aotani, M. D. Thouless and Neil P. Dasgupta**

Supporting Information

Effects of Interfacial Adhesion on Lithium Plating Location in Solid-State Batteries with Carbon Interlayers

Daniel W. Liao[1], *Davy Zeng*[2], *Muzamil Mulla*[1], *Ali Madanchi*[1], *Hiroki Kawakami*[3], *Yuichi Aihara*[3], *Koichiro Aotani*[3], *M. D. Thouless*[1,2], *Neil P. Dasgupta*[1,2]*

[1] Department of Mechanical Engineering, University of Michigan, Ann Arbor, MI, 48109 USA

[2] Department of Materials Science and Engineering, University of Michigan, Ann Arbor, MI, 48109 USA

[3] Nissan Research Center, Nissan Motor Co., Ltd., Natsushima, Yokosuka, Kanagawa 237-8523, Japan

* Corresponding Author. E-mail Address: ndasgupt@umich.edu

Correction for plasticity in the peel test

In the absence of plasticity, and with an inextensible tape, the toughness of an interface, Γ, is related to the peel force, F, in a 180° peel test by the expression[1,2]

$$\Gamma = 2\left(\frac{F}{b}\right) \quad \textbf{(2)}$$

where b is the width of the tape. The assumption of inextensibility is valid for the Kapton tape because the measured values of F are three orders of magnitude less than $2Ebt$, where E is the modulus of the tape (3.6 GPa), and t is the thickness of the tape (25 μm).[1]

However, we noticed that the Kapton® tape generally developed a permanent curl after the peel test. This indicated the need to correct the peel test results to account for the effects of some plastic bending. A schematic of the peel test is shown in Fig. S1; it is the bending moment, M, at the crack tip that induces the plasticity that needs to be considered. The correction that allows the toughness, Γ, to be computed from the peel force F is shown for two thicknesses of Kapton® tape in Fig. S1 and compared to the elastic solution of Eqn. 2. The calculations that give these corrections are detailed below.

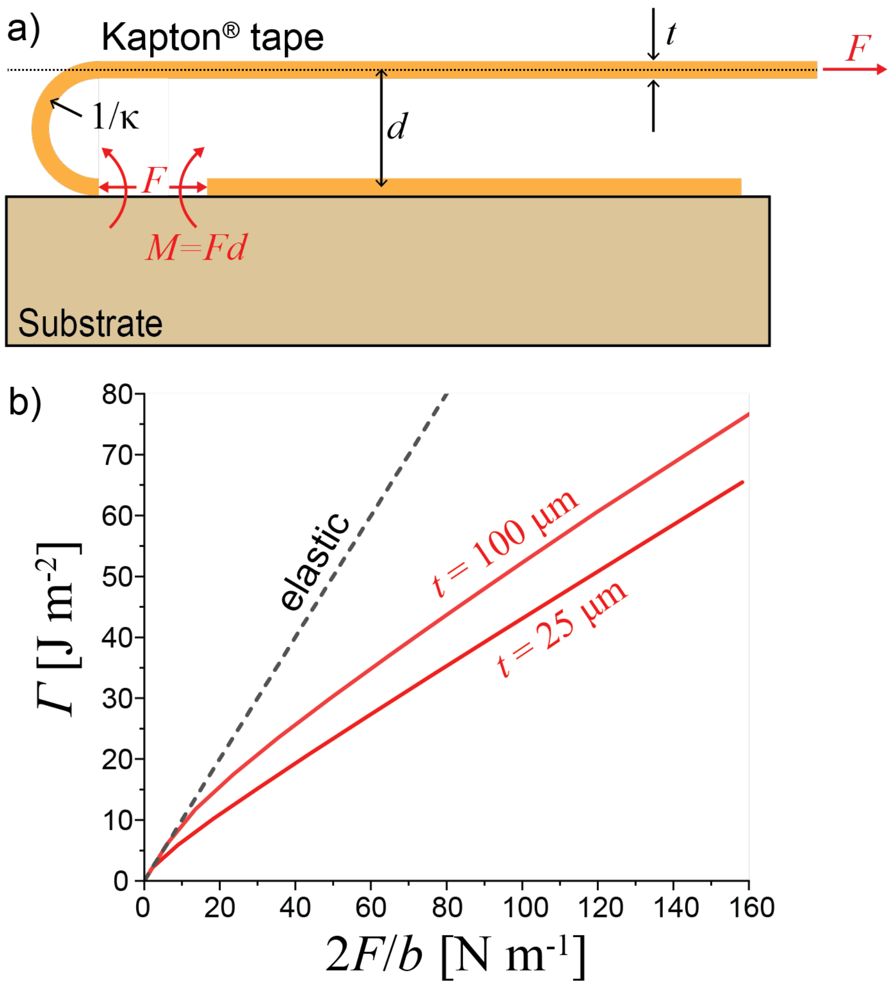

Figure S1: (a) The 180º peel test schematic indicating the location of plastic bending due to the moment at the crack tip. (b) The resulting interfacial toughness values accounting for plastic bending of Kapton® tape with a thickness of 25 or 100 μm and the dash line representing if the tape was taken to be purely within the linear-elastic regime.

If the toughness of the interface is Γ, we can state from thermodynamics that the maximum moment in the tape, $M_{\max}$, (at the crack tip) that will hold a crack in equilibrium is

$$\Gamma b = M_{\max}\kappa_{\max} - \int_0^{\kappa_{\max}} M d\kappa \tag{3}$$

where κ_{max} is the elastic/plastic curvature corresponding to the maximum bending M_{max}. The work dissipated per unit length of crack advance in a 180° peel test is $2F$. This is equal to the total work done during bending (per unit length of tape) minus the elastic energy released upon unloading:

$$2F = M_{max}\kappa_{max} - 6M^2_{max}/Ebt^3 \quad \textbf{(4)}$$

Equations (3) and (4) can be combined to give the required relationship between F, which is measured, and Γ, which is the toughness we are interested in.

In this analysis, we focus only on the effects of the bending strain. We assume that the peel test results in a monotonic loading of the tape, consisting of linear elasticity up to a yield strain of ε_Y, followed by a power-law hardening relationship of the form

$$\sigma = A\varepsilon^n \quad \textbf{(5)}$$

where σ is the axial stress, ε is the axial strain, and A and n are the power law constants. Unloading occurs elastically from the maximum stress reached during power-law hardening. The relationship between the strain and the local curvature, κ, of the centroidal axis (assumed to lie at the center of the tape) is given by

$$\varepsilon = y\kappa \,, \quad \textbf{(6)}$$

where y is the distance from the centroidal axis. The relationship between the moment and the curvature of the tape is

$$M = 2b\int_0^{t/2} \sigma y \, \mathrm{d}y \,, \quad \textbf{(7)}$$

where t is the thickness of the film. The initial portion of the integral follows the linear relationship $M = \kappa E b t^3/12$, up to $M_Y = \kappa_Y E b t^3/12$, where $\kappa_Y = 2\varepsilon_Y/t$. Above this value of curvature, the power-law relationship is followed. Hence, the moment can be expressed in terms of the curvature by

$$M = 2Eb\kappa \int_0^{\varepsilon_Y/\kappa} y^2 dy + 2Ab\kappa^n \int_{\varepsilon_Y}^{t/2} y^{n+1} dy \qquad \textbf{(8)}$$

This results in an equation for M:

$$M = Et^3 b\kappa/12 \quad \text{for} \qquad \kappa \leq 2\varepsilon_Y/t \qquad \textbf{(9)}$$

$$M = \left[\frac{2E\varepsilon_Y^3}{3} - \frac{2A\varepsilon_Y^{n+2}}{n+2}\right] b\kappa^{-2} + \frac{At^{n+2}}{2^{n+1}(n+2)} b\kappa^n \quad \text{for} \qquad 2\varepsilon_Y/t \leq \kappa \leq \kappa_{\max} \qquad \textbf{(10)}$$

We determined that published data[4] for the constitutive properties of Kapton® tape can be described with $n = 0.51$, $A = 381$ MPa and $\varepsilon_Y = 0.01$. Substituting these values into the equation above, with $t = 25$ μm or $t = 100$ μm, results in the relationship between F and Γ shown in Fig. S11.

We then used the relationship for $t = 25$ μm to convert the recorded peel forces into a toughness for the interface. These data are shown in Table S1. Note that no correction for plasticity is needed only for the lowest value of toughness, Γ=1.2 J m^{-2}, when only elastic deformation occurs at the crack tip.

Table S1. Measured peel force and calculated interfacial toughness for amorphous carbon and hard carbon laminated onto an LPSCl solid electrolyte

Interlayer	Lamination Pressure [MPa]	$2F/b$ [N m^{-1}]	Γ [J m^{-2}]
Amorphous Carbon	5	12±2	7±1
	100	16±4	9±2
	200	29±4	15±2
	300	55±6	26±4
	400	96±11	41±5
	600	157±12	65±5
Hard Carbon	100	1±0.5	1±0.5
	200	3±1	3±0.5
	300	11±2	7±1
	400	19±4	11±2
	500	22±6	128±3
	600	33±1	16±1
	800	36±1	18±1

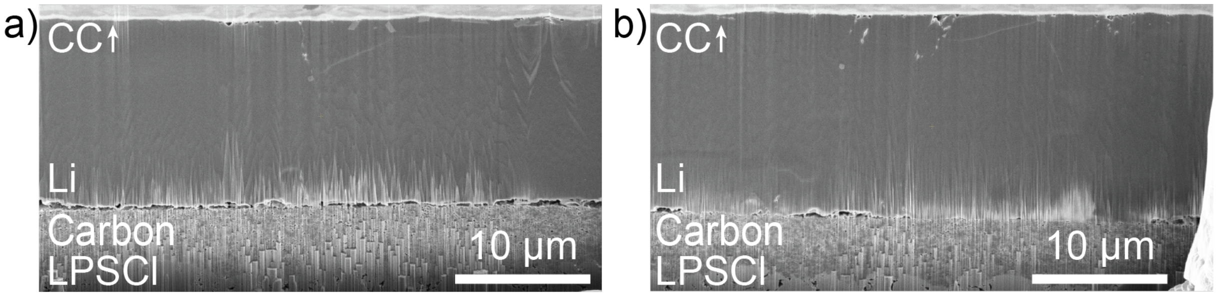

Figure S2: Cross-sectional plasma focused ion beam – scanning electron microscopy (PFIB-SEM) images at two (a-b) different locations for an amorphous carbon interlayer laminated at 400 MPa after plating a capacity of 2.0 mAh cm^{-2} at a current density of 0.1 mA cm^{-2}.

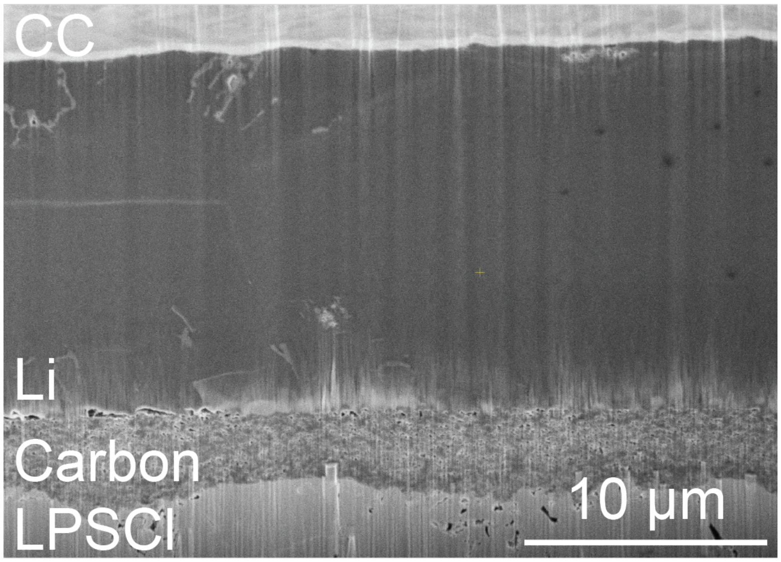

Figure S3: Cross-sectional PFIB-SEM image of an amorphous carbon interlayer laminated at 600 MPa after plating a capacity of 2.0 mAh cm^{-2} at a current density of 0.1 mA cm^{-2}.

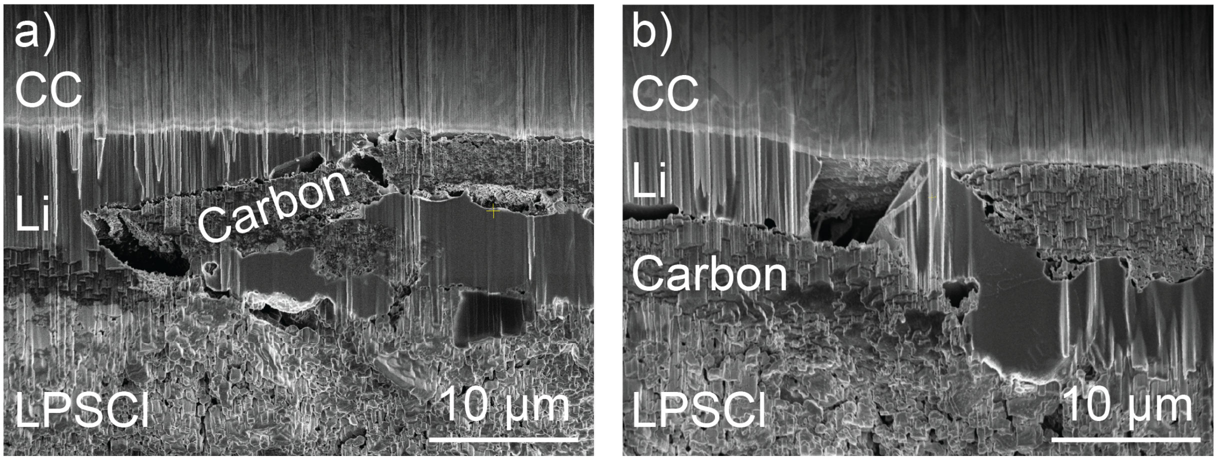

Figure S4: Cross-sectional PFIB-SEM images at two (a-b) different locations of an amorphous carbon interlayer laminated at 100 MPa after plating a capacity of 2.0 mAh cm^{-2} at a current density of 0.1 mA cm^{-2}.

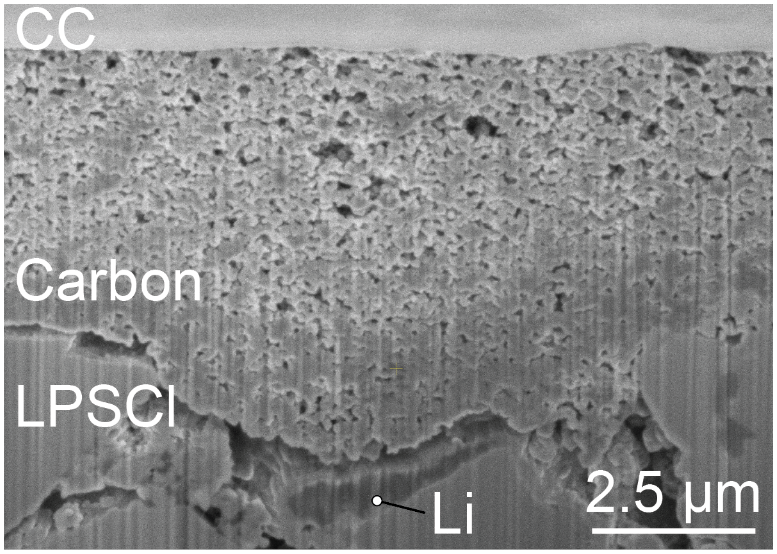

Figure S5: Cross-sectional PFIB-SEM image of an amorphous carbon interlayer laminated at 100 MPa after lithiation to 0 V at 0.01 mA cm^{-2}, and then passing an additional capacity of 0.15 mAh cm^{-2} at a current density of 0.1 mA cm^{-2}. The initial slow charge step was performed to allow for the carbon interlayer to fully lithiate to prevent any solid-state Li insertion of the plated Li into the carbon.

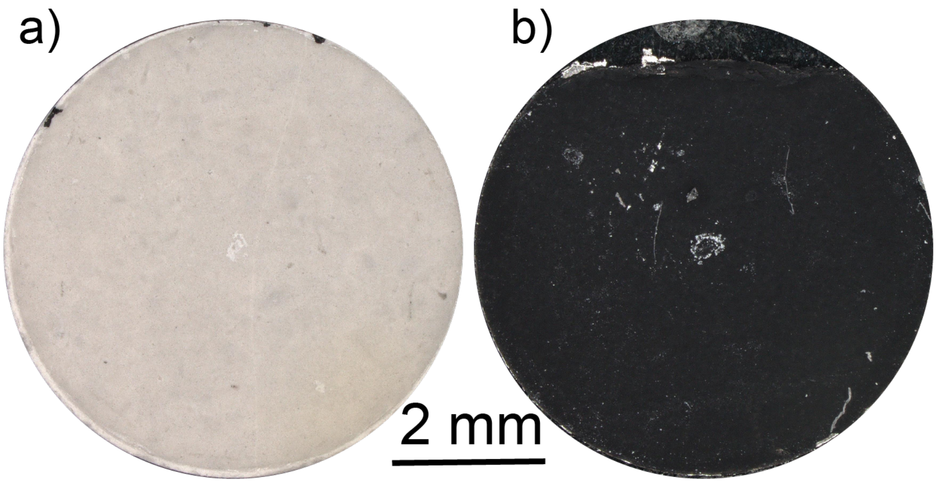

Figure S6: Plan-view optical microscopy images of (a) Li_6PS_5Cl (LPSCl) pellet and (b) as-cast carbon interlayer on stainless-steel CC after applying 5 MPa stack pressure. The carbon interlayer does not transfer onto the solid electrolyte.

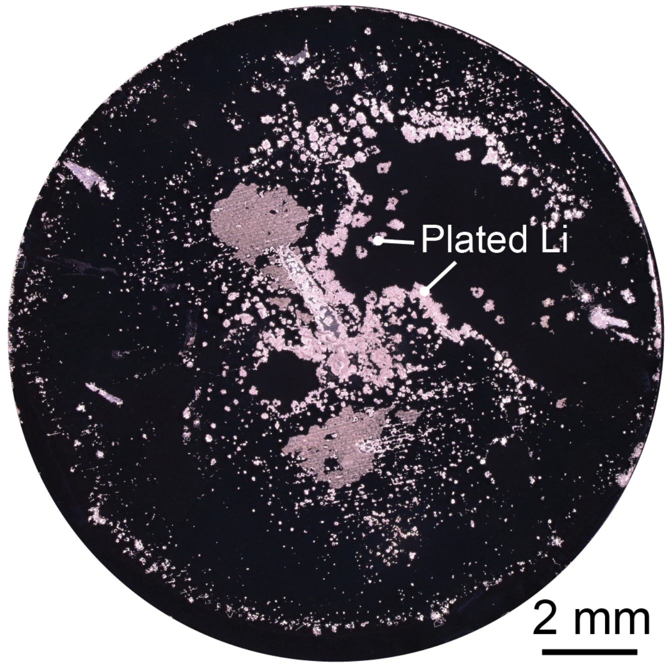

Figure S7: Plan-view optical microscopy image of carbon interlayer surface after plating a capacity of 2 mAh cm^{-2} at 0.1 mA cm^{-2} in a coin cell with a liquid electrolyte. Li plating can be observed on top of the carbon surface that was in contact with the Celgard separator.

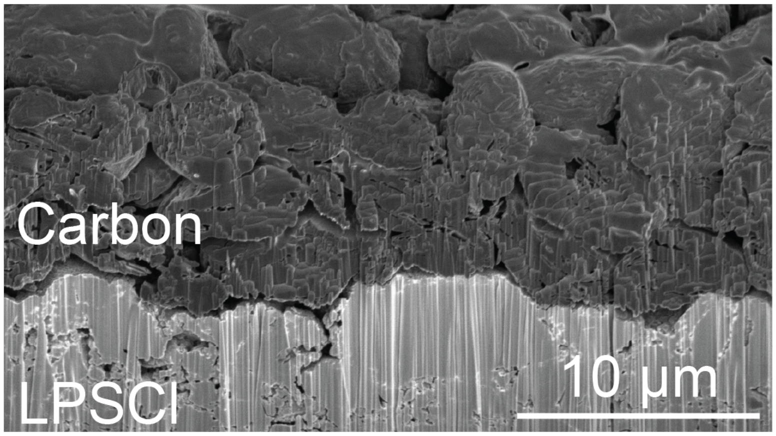

Figure S8: Cross-sectional PFIB-SEM image of a hard carbon interlayer laminated onto an LPSCl pellet at 400 MPa.

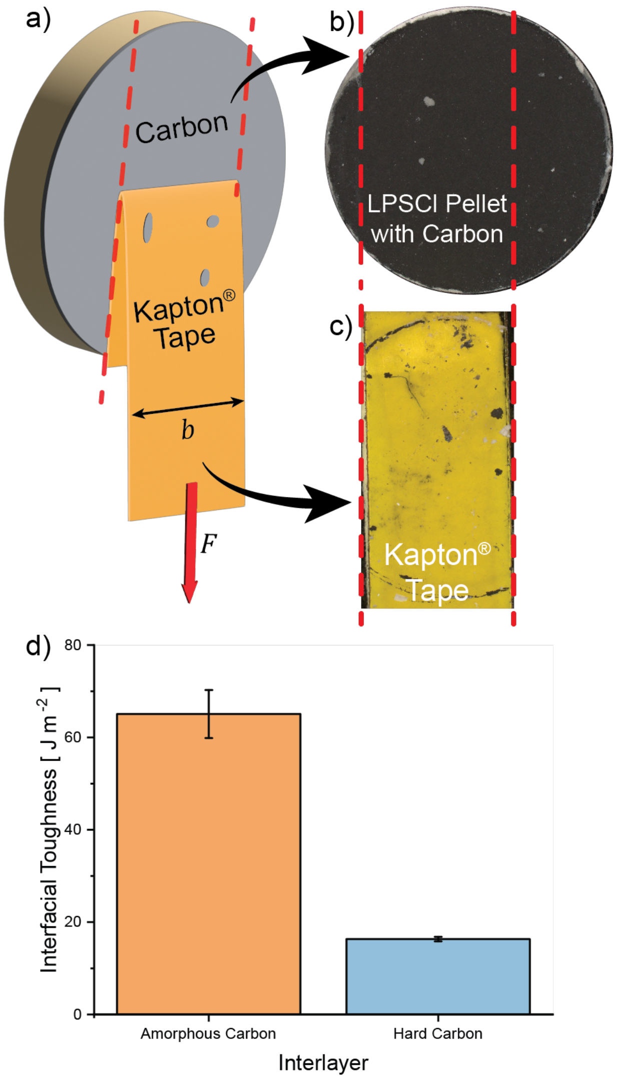

Figure S9: (a) Peel test schematic of Kapton® tape delaminating from the carbon interlayer surface when the interfacial adhesion between carbon and the SE is sufficiently high. Top-down optical microscopy image of the (b) LPSCl pellet with intact carbon interlayer and (c) Kapton® tape after a peel test with amorphous carbon interlayer laminated onto the SE at 600 MPa. (d) The resulting measured interfacial toughness values of the interface between the Kapton® tape and carbon interlayer at a 600 MPa lamination pressure for both amorphous and hard carbon interlayers. Error bars are independent measurements from $n = 3$ samples.

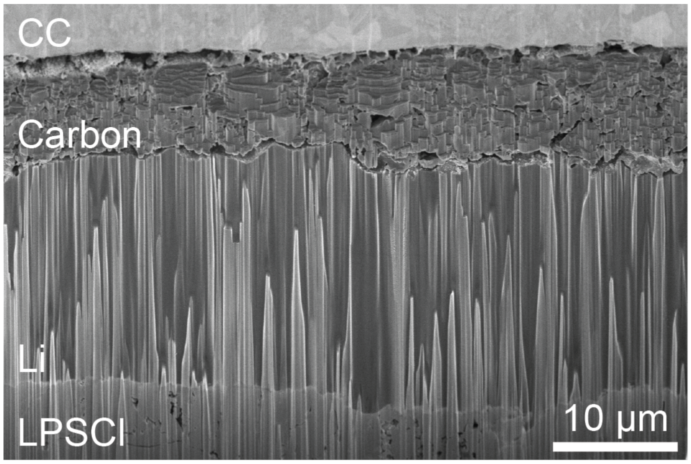

Figure S10: Cross-sectional PFIB-SEM images at on the hard carbon laminated at 400 MPa after plating a capacity of 2.0 mAh cm^{-2} at a current density of 0.1 mA cm^{-2}.

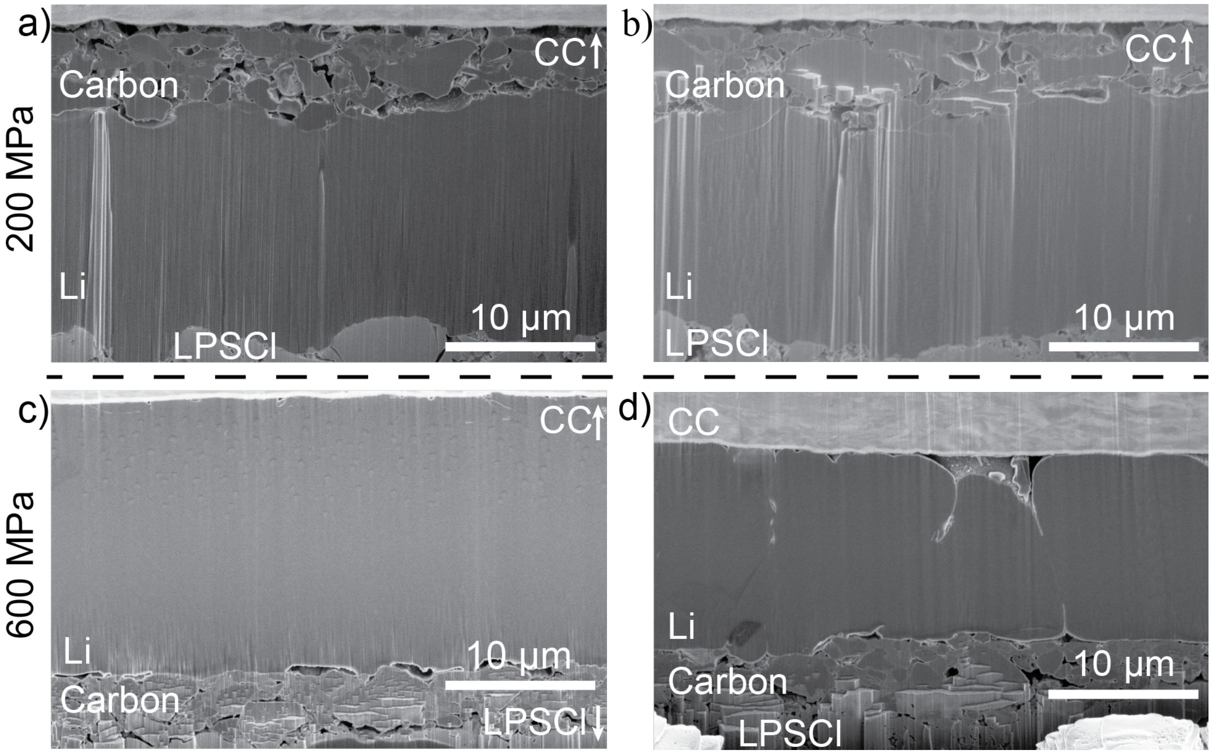

Figure S11: Cross-sectional PFIB-SEM images at two (a-b) different locations for the hard carbon laminated at 200 MPa and (c-d) 600 MPa after plating a capacity of 2.0 mAh cm^{-2} at a current density of 0.1 mA cm^{-2}.

References

1 K. Kendall, *J Phys D Appl Phys*, **1975**, 8, 1449.

2 K. Kendall, *J Phys D Appl Phys*, **1971**, 4, 1186.

3 K. S. Kim and N. Aravas, *Int J Solids Struct*, **1988**, 24, 417–435.

4 D. Y. W. Yu and F. Spaepen, *J Appl Phys*, **2004**, 95, 2991–2997.
